# Lipid Secretion by Parasitic Cells of *Coccidioides* Contributes to Disseminated Disease

**DOI:** 10.3389/fcimb.2021.592826

**Published:** 2021-05-13

**Authors:** Carlos Alberto Peláez-Jaramillo, Maria Del Pilar Jiménez-Alzate, Pedronel Araque-Marin, Chiung-Yu Hung, Natalia Castro-Lopez, Garry T. Cole

**Affiliations:** ^1^ The Biology Department and South Texas Center for Emerging Infectious Diseases, University of Texas at San Antonio, San Antonio, TX, United States; ^2^ Grupo Interdisciplinario de Estudios Moleculares, Chemistry Institute, Faculty of Natural and Exact Sciencess, Medellín, Antioquia, Colombia; ^3^ Grupo Micología Médica, Microbiology and Parasitology Department, School of Medicine, Universidad de Antioquia, Medellín, Antioquia, Colombia; ^4^ School of Life Sciences, EIA University (Universidad Escuela de Ingenieros de Antioquia), Envigado, Antioquia, Colombia

**Keywords:** *Coccidioides*, spherule outer wall, phospholipids, sphingolipids, immunomodulation, fungal lipids, SOW-lipid extract

## Abstract

*Coccidioides* is a soil-borne fungal pathogen and causative agent of a human respiratory disease (coccidioidomycosis) endemic to semi-desert regions of southwestern United States, Mexico, Central and South America. Aerosolized arthroconidia inhaled by the mammalian host first undergo conversion to large parasitic cells (spherules, 80–100 μm diameter) followed by endosporulation, a process by which the contents of spherules give rise to multiple endospores. The latter are released upon rupture of the maternal spherules and establish new foci of lung infection. A novel feature of spherule maturation prior to endosporulation is the secretion of a lipid-rich, membranous cell surface layer shed *in vivo* during growth of the parasitic cells and secretion into liquid culture medium during *in vitro* growth. Chemical analysis of the culture derived spherule outer wall (SOW) fraction showed that it is composed largely of phospholipids and is enriched with saturated fatty acids, including myristic, palmitic, elaidic, oleic, and stearic acid. NMR revealed the presence of monosaccharide- and disaccharide-linked acylglycerols and sphingolipids. The major sphingolipid components are sphingosine and ceramide. Primary neutrophils derived from healthy C57BL/6 and DBA/2 mice incubated with SOW lipids revealed a significant reduction in fungicidal activity against viable *Coccidioides* arthroconidia compared to incubation of neutrophils with arthroconidia alone. Host cell exposure to SOW lipids had no effect on neutrophil viability. Furthermore, C57BL/6 mice that were challenged subcutaneously with *Coccidioides* arthroconidia in the presence of the isolated SOW fraction developed disseminated disease, while control mice challenged with arthroconidia alone by the same route showed no dissemination of infection. We hypothesize that SOW lipids contribute to suppression of inflammatory response to *Coccidioides* infection. Studies are underway to characterize the immunosuppressive mechanism(s) of SOW lipids.

## Introduction

*Coccidioides posadasii* and *Coccidioides immitis* are dimorphic fungal pathogens, the etiologic agents of coccidioidomycosis, a mild to potentially life-threatening respiratory disease. *Coccidioides* spp. is a desert soil-inhabiting fungal pathogen that is found in the southwestern United States and in certain regions of Mexico, Central America, and South America. Despite the genetic diversity between the two species of *Coccidioides* revealed by comparative genomic sequence analyses (Fisher et al., 2001; Sharpton et al., 2009), laboratory studies have shown that they have comparable virulence in mice. *Coccidioides* spp. is a primary pathogen that can cause diseases in both immunocompetent and immunocompromised individuals (Galgiani et al., 2005). Disease onset typically results from inhalation of dry, air-dispersed arthroconidia released by the soilborne saprobic phase of the pathogen. Inhaled arthroconidia of *Coccidioides* spp become hydrated and undergo isotropic growth to form spherule initials (10 to 30 μm diameter) (Cole et al., 2006). These parasitic cells presumably first come in contact with epithelial cells and macrophages in the respiratory tract of the host. These spherules grow isotropically to produce large parasitic cells (60 to >100 μm in diameter) and once the spherule is developed, a surface outer wall (SOW) is produced (Cole et al., 1988). The spherules undergo an elaborate process of endogenous wall growth and cytoplasmic compartmentalization, which culminates in production and the release of a multitude of endospores (each of them 4 to 10 μm in diameter). Endospores are small enough to be disseminated hematogenously, grow and differentiate into a second generation of spherules (Cole et al., 1988). It is suggested that SOW produced is around the endospores released in order to protect them from phagocytosis or killing by the cells of the innate immune response.

Tarbet and Breslau (1953) in their founding studies reported that the walls of mature spherules are rich in “lipid complexes”, which they identified as phospholipids. These authors suggested that the lipid layer of the spherule wall may “resist the diffusion of large molecules”, but “retain certain chemotactic substances within the cell and block or alter chemical interchange between parasite and tissues” of the host. The lipid-rich SOW layer may be a protective barrier that contributes to the survival of the pathogen in host tissue. Frey and Drutz (1986) presented evidence that an extracellular matrix produced by the spherule may partly account for its survival in the presence of leukocytes from healthy donors. The authors suggested that the matrix might impede contact between polymorphonuclear neutrophils and the fungus and somehow reduce the efficiency of host attack against spherules. Release of some of these immunoreactive macromolecules *in vivo* may be attributed to digestive activity by host cells (e.g. polymorphonuclear neutrophils) adjacent to the spherule envelope (Drutz and Huppert, 1983).

The physical properties of membrane lipids in pathogenic fungi have received significant attention in recent years. Studies have shown that lipid microdomains consisting of glycosphingolipids and sterols might serve to concentrate virulence factors (Siafakas et al., 2006; Farnoud et al., 2014), infectivity (Tagliari et al., 2012), and pathogenicity (Singh et al., 2012). Thus, the physical properties of the plasma membrane appear to affect the outcome of the infection (Rella et al., 2016. Certain glycosphingolipids, such as glucosylceramide (GlcCer), have been shown to be involved in the regulation of virulence in fungi affecting plants (Thevissen et al., 2004; Ramamoorthy et al., 2007) and humans (Rittershaus et al., 2006; Singh and Del Poeta, 2011).

The synthesis of GlcCer has been demonstrated in fungi that are pathogenic to humans, such as *Cryptococcus neoformans* (*C. neoformans)* (Hogan et al., 1996), *Candida albicans* (*C. albicans*) (Leipelt et al., 2001), *Aspergillus fumigatus* (*A. fumigatus*) (Levery et al., 2002), *Histoplasma capsulatum* (*H. capsulatum*) (Klimpel and Goldman, 1988), *Paracoccidioides brasiliensis* (*P. brasiliensis*) (San-Blas and San-Blas, 1977), and *Sporotrix schenckii* (*S. schenckii*) (Toledo et al., 2000). Other studies have suggested a role for GlcCer in the regulation of fungal growth and pathogenesis. For instance, in *C. neoformans* GlcCer is mainly localized in the cell wall and mostly accumulates at the budding site of dividing cells (Rittershaus et al., 2006). Interestingly, antibodies against *C. neoformans* GlcCer produced by patients affected with cryptococcosis inhibit budding and division of *C. neoformans* cells grown *in vitro* (Rodrigues et al., 2000) as well as differentiation and germ-tube formation of *Pseudallescheria boydii* (*P. boydii*) and *C. albicans* (Shimamura, 2012). Additionally, production of antibodies against fungal glycolipids has been demonstrated in patients with paracoccidioidomycosis (Bertini et al., 2007). In other fungi, disruption of the GlcCer biosynthetic pathway altered spore germination, hyphal development, and fungal growth (Levery et al., 2002). Monoclonal antibodies against fungal GlcCer have been produced, and interestingly, these antibodies protected mice against lethal cryptococosis (Rodrigues et al., 2007), and also the treatment with anti-GlcCer antibody enhanced macrophage function against the fungus *Fonsecaea pedrosoi* (*F. pedrosoi*) (Nimrichter et al., 2004). Taken together, these studies suggest an important role of GlcCer in fungal cell growth and differentiation. Furthermore, GlcCer might also be implicated in the regulation of fungal virulence.

Lipid composition of fungal cell wall has been characterized for several species. Studies of the parasitic phase (yeast) of the dimorphic fungus *P. brasiliensis* have identified 49 phospholipid including phosphatidylcholine, phosphatidylethanolamine, phosphatidylserine, phosphatidylglycerol, phosphatidylinositol, and phosphatidic acid (Longo et al., 2013). Among the fatty acids, C18:1 and C18:2 were the most abundant. The prevalent glycolipid species was Hex-C18:0-OH/d19:2-Cer, although other minor glycolipid species were also detected. The most abundant sterol was brassicasterol (Longo et al., 2013).

In the present study, the chemical analysis of the lipids extracted from the spherule outer wall (SOW) fraction of *Coccidioides posadasii* showed that they are composed largely of phospholipids, enriched with saturated fatty acids and the major sphingolipid components are sphingosine and ceramide. A neutrophil killing assay and a murine model of coccidioidomycosis were also explored to characterize the biological effect of SOW lipids in immune function.

## Materials And Methods

### *Coccidioides* Cultures and Spherule Staining

*Coccidioides posadasii* isolate C735 is a virulent clinical isolate. Hyphal growth of *Coccidioides* spp were cultured on glucose-yeast extract (GYE) agar plates at 25°C for 4 weeks to produce arthroconidia that were used to produce spherules in Converse medium using 250 ml Erlenmeyer flasks with rubber stoppers. Each culture flask contained 1–5 × 10^7^ arthroconidia in 100 ml of Converse medium that was purged with a medical grade gas mixture (20% CO_2_ and 80% air) for 3 min and incubated in a CO_2_ incubator for 5–14 days at 39°C, 10% CO_2_ with shaking (Cole et al., 1985). The flasks were purged with C0_2_-air every 2 days after inoculation. Production of the sloughing, membranous SOW was monitored by phase-contrast microscopy. All steps of the inoculation and subsequent isolation procedure were performed in a Biosafety level 3 laboratory at University of Texas at San Antonio.

Fungal cells were labeled with a florescent dye cocktail containing 0.4 mg/ml Calcofluor White (CFW; Sigma, St. Louis, MO) and 5% FM^™^4-64FX (Thermo-Fisher Scientific, Waltham, MA), which bind to cell wall and lipophilic membrane, respectively. Spherules were washed twice with PBS and resuspended in 2% PFA. Cell images were acquired with an Amines ImageStream MKII cytometer and analyzed using IDEAS^®^ software.

### SOW Isolation and Lipid Extraction

SOW was isolated from parasitic culture of *Coccidioides* in Converse medium as previously reported (Cole et al., 1988). Hexane was added to SOW, and an aliquot of the SOW fraction was subjected to sterility tests on GYE culture plates. Sterile SOW fractions were then taken to BSL2 laboratory for lipid extraction.

Two extraction methods were performed, a one-step method with hexane alone and a sequential extract method using three solvents (hexane, chloroform, and methanol) with increased polarity (JT Baker. ThermoFisher Scientific Inc.). The extraction procedures were conducted with a percolating Soxhlet extractor. The extracts were concentrated by rotary evaporation and then further dried under a nitrogen gas stream. The absence of the glycoprotein in the SOW lipid fraction was confirmed by immunoblot analysis with anti-SOWgp glycoprotein serum (Hung et al., 2002) and the Bradford method to estimate the protein content (Bradford, 1976).

### Characterization of Lipid Extracts of SOW by Thin Layer Chromatography

The qualitative characterization of lipids extracted from SOW was performed by one- and two-dimensional thin layer chromatography (TLC) using silica gel 60 F254 (Merck^®^, USA) plates. The lipid bands/spots on silica plates were revealed with iodine and visualized under an UV-light. The retention factor (Rf) was used to compare and identify the SOW lipids. The Rf value is equal to the distance traveled by the lipid divided by the distance traveled by the solvent front, both measured from the origin.

### Gas Chromatography Analysis of SOW Fatty Acids

Fatty acids were extracted from the lipid fractions with 0.5 N NaOH and then methylated with 20% boron trifluoride (BF3) using a standard protocol (Ackman, 1998; Ichihara and Fukubayashi, 2010). The methylated fatty acids were purified in n-Heptane and dehydrated using anhydrous sodium sulfate. Finally, the sample was filtered and injected into a gas chromatograph (Agilent 6890N) that is equipped with a flame ionization detector (FID). The samples were separated using a capillary column (DB23). Data was acquired and analyzed using Chemstation software.

### UPHPLC-MS/MS Analysis

Phospholipids and glycolipids were characterized by electrospray ionization tandem mass spectrometry (ESI-MS/MS) on a linear ion-trap mass spectrometer system using a ACQUITY UPLC^®^ BEH C18 column (Xevo G2-XS QTOF Quadrupole Time-of-Flight Mass Spectrometry; Waters Corporation, Milford, MA. EEUU). The mobile phase contained a mixture of formic acid and acetonitrile solution, at a flow rate of 0.300 ml/min and ionization source ESI positive detector was used to acquire the data. Samples were dissolved in 10 mM LiOH/methanol with 2.5 mM phosphatidylcholine (C11:0/C11:0-PC) as an internal standard. Full-scan spectra were collected at the 500–1,000 m/z range, and samples were subjected to total-ion mapping (TIM) [2 a.m.u. isolation width; pulsed-Q dissociation (PQD) to 29% normalized collision energy; activation Q of 0.7; and activation time of 0.1 ms]. Methylated glycolipids were dissolved in methanol and analyzed as described for phospholipids with some modifications (acquisition at the 500–2000 m/z range; PQD to 32% normalized collision energy). Spectra from both phospholipids and glycolipids were analyzed manually according to Pulfer and Murphy, 2003.

### Chemical Structure Analysis of SOW Lipids Using FTIR and NMR

Methanol lipid extracts of SOW were suspended in 20 mM HEPES buffer (pH 7.4) at molar ratios from 1:0.0 to 1:1.0 for infrared spectroscopic measurements using an IFS-55 spectrometer (Bruker, Karlsruhe, Germany). Samples were placed in a CaF2 cuvette with a 12.5 μm Teflon spacer. Consecutive heating scans were performed automatically from 10 to 70°C with a heating rate of 0.6°C min^-1^. Every 3°C, 200 interferograms were accumulated, apodized, Fourier transformed, and converted to absorbance spectra. The peak position of the asymmetric stretching vibration of the methylene band versus (CH_2_) sensitive marker lipid order was plotted versus temperature. Phase transition temperatures were derived by determination of the maximum of the first derivative of the heating scans. For measurement of hydrated lipids, samples were spread on an attenuated total reflectance (ATR) ZnSe crystal and free water was evaporated under a stream of N_2_. Vibrational bands from the interface region (1,700–1,750 cm^−1^), amide I (1,600–1,700 cm^−1^), and head groups (1,000–1,300 cm^−1^) were analyzed. The instrumental wave number resolution was better than 0.02 cm^−1^; the wave number reproducibility in repeated scans was better than 0.1 cm^−1^.

The ^1^H- and ^13^C-NMR and two-dimensional spectra were obtained in an AMX300 spectrometer (Bruker BioSpin GmbH, Rheinstetten, Germany) operating at 300 MHz for ^1^H and 75.0 for ^13^C using CDC^l3^ or dimethyl sulfoxide d6. Shifts are reported in *δ* units (ppm) and coupling constants (*J*) in Hz.

### Polymorphonuclear Neutrophil Isolation and Killing Assay

Inbred C56BL/6 and DBA/2J mice were obtained from the National Cancer Institute/Charles River Laboratories. Mice were housed in a specific-pathogen free animal facility at UTSA and handled according to guidelines approved by the IACUC at UTSA. At 8 to 12 weeks of age, sex-matched mice were relocated into an ABSL3 laboratory before experimentation.

Polymorphonuclear neutrophils (PMN*Ф*s) were harvested from peritoneal exudates of C57BL/6 and DBA/2J mice that were intraperitoneally injected with 4% thioglycollate at 4 h post injection. PMN*Ф*s were further enriched using a Ficoll-Paque density gradient and incubated with *C. posadasii* arthroconidia (MOI: 5:1) plus an indicated concentration of the SOW lipid methanol extracts for 4 h at 35°C, 5% CO_2_. PMN*Ф*s incubated with arthroconidia alone were used as a control. Percentages of killing were determined by serial dilution on GYE agar plates of the mixtures as previously described (Gonzalez et al., 2011). Assays of killing efficiency were repeated in three separate experiments.

### Subcutaneous Challenge and Treatment With SOW Lipids

Hair on an area of the posterior quadrant of the abdomen (approximately 2 by 2 cm) was removed and swabbed with 70% ethanol. Mice were challenged subcutaneously (s.c.) with 5 × 10^4^ viable arthroconidia of *C. posadasii* isolate C735 suspended in 100 μl PBS on the posterior border of the hairless abdominal region as previously described (Hung et al., 2016). Arthroconidia inoculation resulted in a small raised skin inflammation (≈2 mm), which dissipated within 24 h post-challenge. The subcutaneous injection of 5,000 μg/ml of SOW lipids was done at days 0, 4, 8, 12, and 16 after *Coccidioides* infection. The fungal burden in infected hypodermal tissue, which included visible abscesses and adjacent draining lymph nodes, was determined at the indicated days post-challenge by plating serial dilutions of the tissue homogenates on GYE agar containing 50 μg/ml chloramphenicol as described previously (Xue et al., 2009). The skin abscesses and adjacent draining lymph nodes were radically enlarged and fused together in most of the infected mice at 9 and 20 days post-challenge (dpc). Thus, both tissues were combined for CFU determination. The fungal burdens in skin, lung and spleen homogenates were determined in the same manner. The number of CFU of *Coccidioides* was expressed on a log scale and reported for individual mice of each group as previously described (Xue et al., 2009).

### Statistical Analysis

The Mann–Whitney U test was used to analyze CFUs and cell numbers as previously described (Xue et al., 2009). A *P*-value of <0.05 was considered statistically significant. The killing percentage of arthroconidia by PMN*Ф*s treated with SOW lipids was analyzed with one-way ANOVA. The GraphPad software version 6.0a was used for the statistical analysis.

## Results

### *Coccidioides* SOW Mainly Contains Lipids

**Figure 1:**
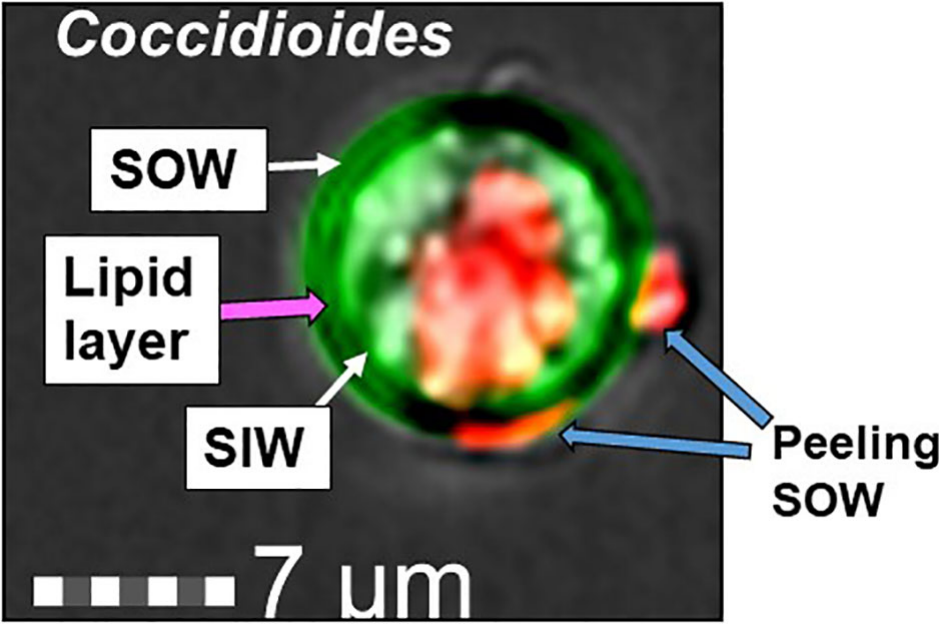
Image of a *Coccidioide*s spherule labeled with Calcofluor White (green) and FM^™^4-64FX (red). A thin greenish yellow layer visible between the spherule outer and inner wall represents colocation of SOW lipids and fungal cell wall chitin/glucan component. The lipophilic probe, FM^™^4-64FX bound to a central vacuole (V) and peeling SOW.

**Figure 2:**
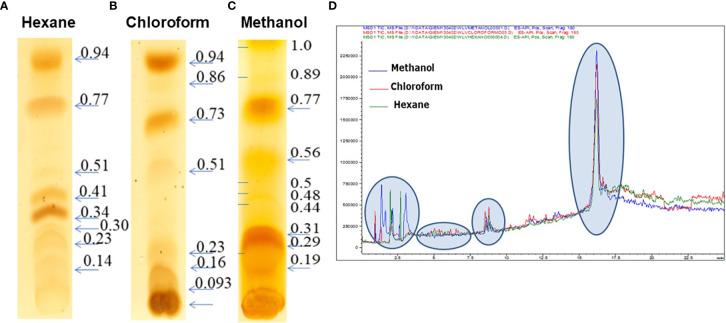
TLC analysis of extracts of SOW lipids with hexane **(A)**, chloroform **(B)** and methanol **(C)** and UPHPLC chromatography of the SOW lipid extracts separated with hexane, chloroform, and methanol **(D)**. TLC separation was carried out with a mixture solvent containing eluent phase as hexane: chloroform 1:2.

*C. posadasii* consistently produces and sheds membranous SOW that is accumulated in the Converse medium *in vitro*. Image analysis of spherules revealed that there is a lipid layer labeled with a lipophilic dye, FM™4-64FX between the spherule outer and the inner wall (SOW & SIW; the greenish yellow layer in **Figure 1**). Notably, the peeling SOW also bound well to FM™4-64FX. Cross examination of the lyophilized SOW extracts showed light-weighted and fiber-like powder appearance (Cole et al., 1988). Approximately, 67% of SOW dry weight was soluble in organic solvents including 53.2% in methanol, 9.9% in chloroform, and 3.9% in hexane, respectively. These fractions did not contain SOWgp protein, a major GPI-anchored antigen located on SOW, as it was not detected in any of the solvent extracts by Western blot analysis with a SOWgp-specific serum (**Supplemental Figure S1**). TLC separation of the lipid extracts revealed that the major compounds were lipids with Rf values at 0.19–0.31, 0.77, and 1.0 in the methanol extract; Rf values at 0.16, 0.73, and 0.94 in the chloroform extract, and 0.34, 0.41, 0.77, and 0.94 in hexane, respectively (**Figures 2A** **–C**). Despite lipid compositions varied in these three extracts, the major lipids were apparently present in all three extracts with most abundant in the methanol extract. Further analysis of lipid extracts using Ultra High Performance Liquid Chromatography (UHPLC) revealed that the spectra of these three solvent extracts were almost overplayed (**Figure 2D**), suggests the methanol extract contained representative SOW lipid species. Hence, we focused on chemical and biological analysis of the methanol extract.

### *Coccidioides* SOW Lipids Consist of Both Phospholipids and Sphingolipids

The major lipid peak (circled in **Figure 2D**; ~17 min) in the methanol extract was subjected to mass spectroscopy analysis (UHPLC-MS/MS). A total of seven phospholipids and six sphingolipids were identified (**Table 1**). GCxGC-TOF analysis of FAMES derived from the methanol extract revealed that the SOW lipids contained mainly saturated fatty acids (30.7%) and only trace amounts of unsaturated molecules (0.17%; **Table 2**). The major fatty acids were palmitic acid (C16:0) and stearic acid (18:0).

**Table 1:** Major lipids identified in the methanol extract of SOW.

Lipids	Fatty acids	Observed mass (*m/z*)	Predicated Mass(+modification)
Phosphatidylethanolamine (PE)	C18:2	493.5	494.6 (M + NH4^+^)
	C14:0	486.1	484.6 (M + NH4^+^)
	C18:0	479.0	481.6 (M^+^)
	C18:1	484.4	480.6 (M + H^+^)
Phosphatidic acid (PA)	C20:0	480.4	482.6 (M + NH4^+^)
	C12:0	375.1	375.3 (M + Na^+^)
Phosphatidylglycerol (PG)	C12:0	486.1	487.6 (M + NH4^+^)
			
Sphingosine (C_18_-*α*-OH-Δ8)		323.4	333.5 (M + NH4^+^)
Sphingosine-phosphate (C_18_-*α*-OH-Δ8)		437.3	432.5 (M + Na^+^)
Sphingomyelin-phosphocholine (C_18_-*α*-OH-Δ4, Δ8, C9-methyl)	C14:0	675.7	673.7 (M + H^+^)
Sphingomyelin-phosphoethanolamine (C_18_-α-OH-Δ4, Δ8, C9-methyl)	C14:0	437.4	435.6 (M + H^+^)
Dihexosylceramide (C_18_-*s*-OH-Δ4)	C14:0	900.7	904.2 (M^+^)
	C16:0	868.3	875.2.3 (M^+^)

**Table 2:** Fatty acid composition of SOW lipids.

Saturated fatty acids	%
Decanoic acid, (C10:0)	0.2
Dodecanoic acid (C12:0)	2.1
Myristic acid (C14:0)	1.4
Pentadecanoic acid (C15:0)	0.2
Palmitic acid (C16:0)	21.2
Heptadecanoic acid (C17:0)	0.5
Stearic acid (C18:0)	3.6
Arachidic acid (C20:0)	0.3
Heneicosanoic acid (C21:0)	0.1
Behenic acid (C22:0)	0.7
Tricosanoic acid (C23:0)	0.2
Lignoceric acid (C24:0)	0.3
**Subtotal**	**30.7**
**Unsaturated fatty acids**	**%**
Elaidic acid (C18:1n9t)	0.12
Oleic acid (C18:1n9c)	
Heneicosanoic acid	0.1
Linolelaidic acid (C18:2n6t)	0.04
Linoleic acid (C18:2n6c)	
**Subtotal**	**0.17**
**Ratio of Unsaturated/Saturated**	**0.0055**

The bold font was used to highlight the values of subtotals of saturated, unsaturated and the ratio between them.

**Figure 3:**
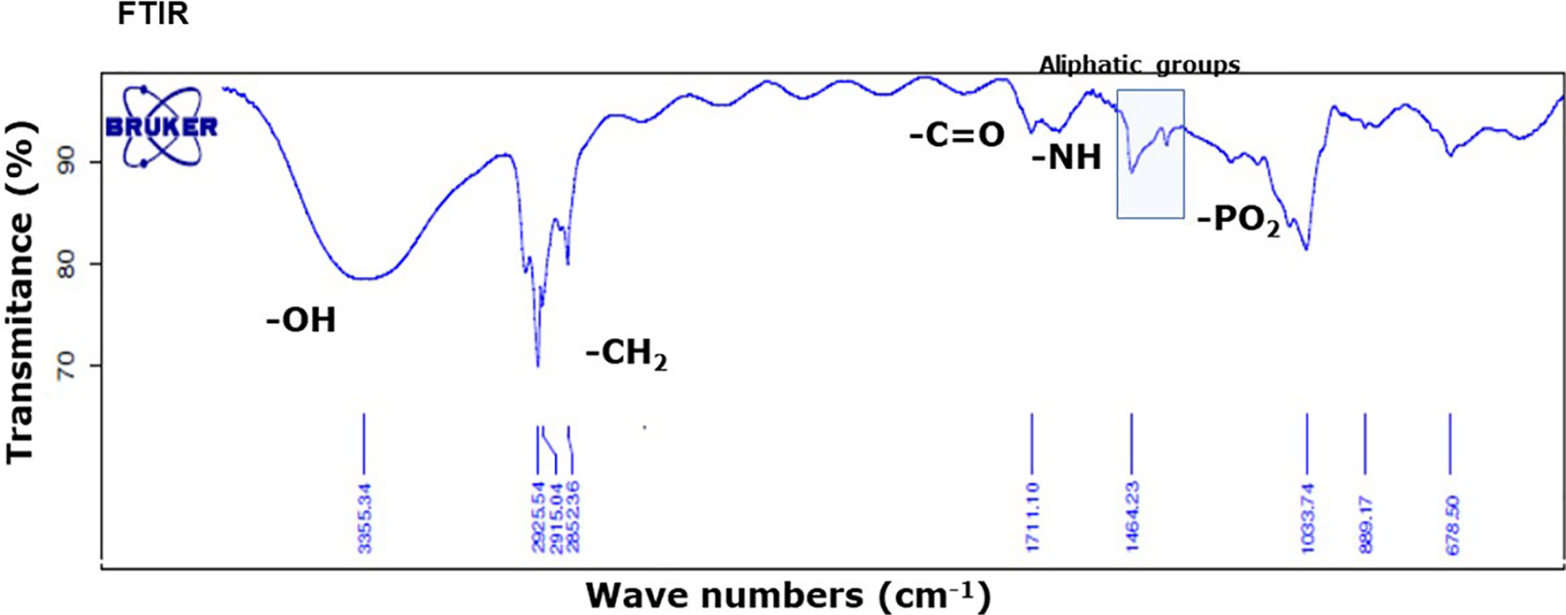
FTIR absorption spectrum of SOW lipids in the methanol extract shows the presence of contribution functional groups (OH, CH_2_, CO, NH, and PO_2_) that are labeled at the corresponding spectral positions.

Infrared (FTIR) analysis of the methanol extract allowed confirmation of the signals of substituents more representatives (**Figure 3**). The signals at 3,364, 2,900, 1,711, 1,641, 1,464, and 1,378 indicate the presence of -OH group linked to alcohols, -CH2 group in aliphatic compounds, -C=O group in fatty acids or esterified compounds, -NH amine group in sphingolipids and phosphate groups in phospholipids. These results were in agreement with the TLC and UHLPC/MS studies (**Figure 2** and **Table 1**). These data suggest that SOW lipids consist of phospholipids and sphingolipids.

**Figure 4:**
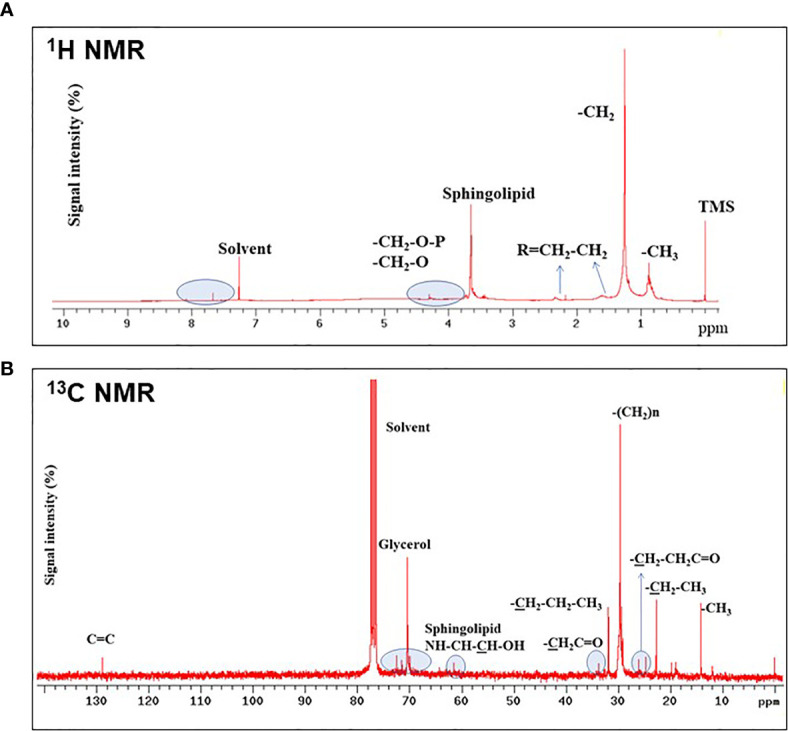
^1^H- **(A)** and ^13^C-NMR **(B)** analyses of SOW lipids in the methanol extract. Contributions from specific functional groups are labeled at the corresponding spectral positions.

Sphingolipids of SOW lipids were further confirmed using both ^1^H- and ^13^C-NMR analyses. ^1^H-NMR analysis showed the presence of protons adjacent at phosphate groups (-CH2-O-P), protons adjacent to oxygens (-CH2-O), methylene adjacent to double- bonds (R=CH2-CH2) or carbon *α* (**Figure 4A**). Additionally, ^13^C-NMR analysis showed the presence of both glycerol and sphingosine (**Figure 4B**). Taken together, SOW lipids consist of fatty acids, triacylclycerols, phospholipids (PA, PE and PG), and sphingolipids including sphingosine chain without a fatty acid and ceramides with a fatty acid side chain.

### SOW Lipids Suppress Neutrophil Killing of *Coccidioides* Arthroconidia *In Vitro*

**Figure 5:**
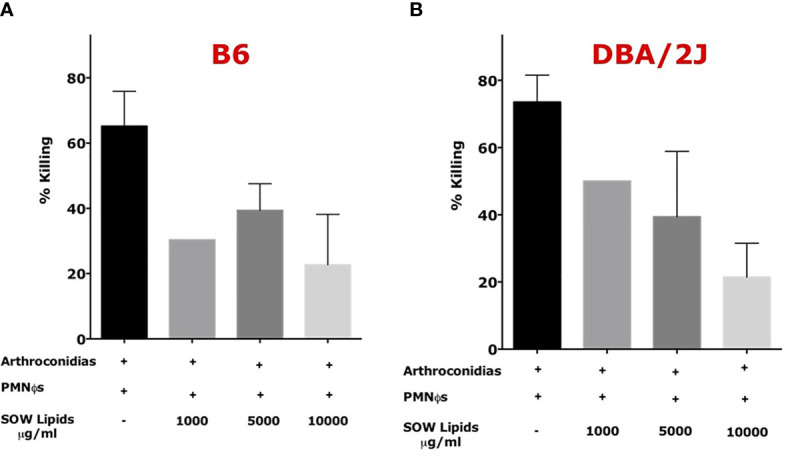
SOW lipid treatment of murine neutrophils (PMN*Ф*s) resulted in loss of killing efficiency of *Coccidioides* arthroconidia. PMN*Ф*s were incubated with *Coccidioides* arthroconidia for 4 h in the presence of SOW lipids at an indicated concentration from 1,000 to 10,000 µg/ml. Bar data show the mean percentage of *Coccidioides* arthroconidia killed for PMN*Ф*s isolated from both C57BL/6 **(A)** and DBA/J mice **(B)**. Representative results (mean ± SD for *n* = 3 technical replicates) per treatment from one of three independent experiments are shown. Killing percentage is presented as mean killing relative to vehicle-treated cells (normalized to 100%). Statistical analysis was done with one-way ANOVA.

Peritoneal neutrophils were isolated from C57BL/6 and DBA/2J mice after eliciting with 4% thioglycolate. Viability and purity of the isolated were assessed as shown in **Table S2**. The purified PMN*Ф*s were capable of killing 60–80% of *Coccidioides* arthroconidia after 4 h incubation. The PMN*Ф*s incubated with an indicated concentration of SOW lipids at 1,000, 5,000, and 10,000 μg/ml significantly decrease the killing activity of *Coccidioides* arthroconidia from ~20 to 50% for B6 and DBA/2J mice, respectively (**Figures 5A, B**).

The difference between different treatments was not statistically significative. The viability of SOW lipids treated PMN*Ф*s were evaluated by flow cytometry analysis after staining with carboxyfluorescein diacetate (CFDA) and Annexin V for detecting apoptotic cells. The results confirmed that PMN*Ф*s were viable after incubating with SOW lipids even at the highest concentration (these results are presented in **Supplemental Figure S3** and **Table S3**). These results suggest that SOW lipids suppress PMN*Ф*s killing functions without impacting viability of these phagocytes.

### SOW Lipids Suppress Protective Immunity Against *Coccidioides* Infection

**Figure 6:**
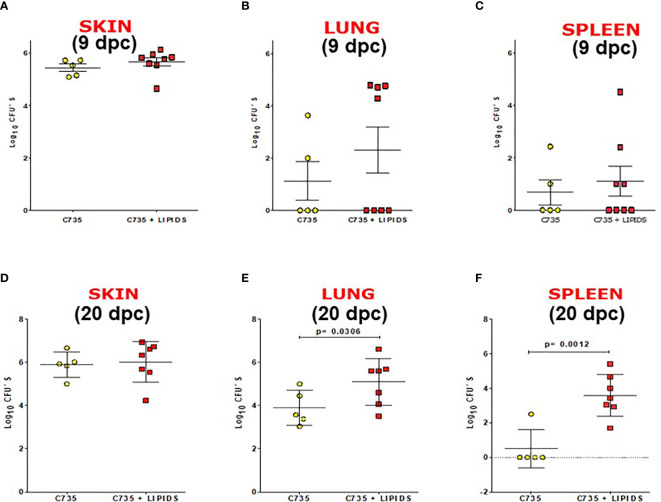
SOW lipids suppress protective immunity against a subcutaneous infection with *Coccidioides.* Comparison of fungal burden in the subcutaneous infection site **(A, D)** lungs **(B, E)** and spleen **(C, F)** of SOW lipid-treated and mock mice at 9 and 20 days post-challenge. The median CFUs (log10) are indicated by horizontal lines inside each group. A statistically significant difference was revealed between the fungal burden in the lungs and spleen of treated and mock mice at 20 dpc.

*In vivo* impact of SOW lipids was evaluated using a murine model of subcutaneous coccidioidomycosis (Hung et al., 2016). A group of mice was treated with 5,000 μg/ml of SOW lipids by the same subcutaneous route at 0, 4, 8, 12, and 16 days post-challenge. Mice were injected with PBS as controls. Both groups of mice were sacrificed at 9 and 20 days post-challenge for evaluating fungal burden (CFUs). The CFU´s recovered from the injection site (skin) at 9 and 20 days post-challenge are shown in **Figures 6A**, **D**, respectively. At 9 days post-challenge, there was a trend of increased fungal burden in the lungs and spleen of the mice that were treated with SOW lipids, albeit that was not statistically significant (**Figures 6B, C** **)**. Interestingly, the mice treated with a total dose of 25,000 μg/ml of SOW lipids and euthanized 20 days post-challenge showed significantly increased amounts of CFUs in their lungs compared to the mice injected with PBS alone (**Figure 6E**; 5.5 Log_10_ versus 4.0 Log_10_; Mann–Whitney U test *p* = 0.0306). Furthermore, the SOW lipid-treated mice also significantly increased fungal dissemination to the spleen (**Figure 6F**; *p* = 0.0012), while only one mouse of the lipid non-treated group had detectable fungal burden in the spleen. These results suggested that SOW lipids suppress protective immunity of mice against subcutaneous *Coccidioides* infection.

## Discussion

Coccidioidal SOW is a unique structure of fungal cell surface among medically important fungal pathogens. In 1953, Tarbet and Breslau demonstrated that *Coccidioides* spherules were positive for the Baker acid-hematin test, a technique to detect phospholipids (Tarbet and Breslau, 1953). Spherules from all stages of parasitic growth are tested positive for surface phospholipids, except endospores. The thickness of the phospholipid layer was seen to be proportional to the size of spherules. The lipid bilayer (tripartite) structure was also shown in electron microscopy examination of SOW that was labeled with osmium tetroxide (OsO4) to enhance the visibility of lipoids (Figure 1 in Cole et al., 1988; Figure 8B in Hung et al., 2002). SOW consists of multiple layers of the bilayer tripartite structures interwoven together with OsO4-negative layers that presumably are made of polysaccharides. Lipids are enriched in the inner leaflets of SOW. When SOW is peeled away from the spherule surface the lipids are exposed and readily bound to lipophilic FM^™^4-64FX dye (**Figure 1A**). Qualitative and quantitative differences in lipid contents are found between fungal species, culture conditions and specific cell structure components including fungal cell wall, whole cells, mycelial wall, spore wall and yeast wall (Longo et al., 2013; Feofilova et al, 2015). Fungal cell wall is thought to mainly compose of glycoproteins and polysaccharides such as *α*- and *β*-linked glucan, chitin, and other sugar polymers. Surprisingly, we found that lipids constitute over 67% of SOW dry weight and they are the major chemical components of spherule outer wall.

SOW is shed into culture media in large quantities once the fungus has undergone a transformation process to spherules in the parasitic phase growth of *Coccidioides* (Cole et al., 1988). SOW is accumulated in the parasitic cultures using a chemically defined Converse medium that only contains glucose as carbon source, N-Z amine and ammonia acetate as nitrogen source, phosphate salts and trace amounts of metals (Converse, 1956 and 1957; Levine, 1962). Spherules in the murine lungs also shed SOW which can be engulfed by adjacent host phagocytic cells. Studies of the chemical compositions and biogenesis of SOW are fundamental steps to learn the biological functions.

SOW is harvested using a stepwise, differential centrifugation method that produces over 50 mg of SOW per litter of spherule cultures at day 7 post inoculation (Cole et al., 1988). We have tested three solvents with increased polarity (hexane, chloroform and methanol) to extract neutral lipids, phospholipids and glycolipids of SOW, respectively. The yield of SOW lipids has been improved by using a percolation Soxhlet system compared to regular sonication method. TLC analysis reveals that there are relatively high portions of non-polar lipids (Rf valve > 0.7) compared to chloroform and methanol extracts, while all lipid types are apparently present in all three solvents. For chemical structure characterization, we have decided to analyze the methanol extract that had the highest lipid yield among the three extracts.

Composition and chemical structures of *Coccidioides* SOW lipids were analyzed using UHPLC-MS/MS, GC-FID, GCxGC-MS, FT-IR and NMR (both ^1^H and ^13^C spectra). SOW lipids include free fatty acids, triacylglycerides, steroids, phospholipids, and sphingolipids. Although these lipid molecules are common for many fungal pathogens and mammalian bilayer membrane, they constitute a specific profile in SOW lipids. SOW lipids are enriched in phospholipids including phosphatidylethanolamine (PE), phosphatidic acid (PA) and phosphatidylglycerol (PG) (**Table 1**). Surprisingly, phosphatidylcholine (PC), an abundant phospholipid of the cell wall of *Paracoccidioides brasiliensis* and other filamentous fungi is not detected (Dembitsky and Pechenkina, 1991; Longo et al., 2013). Interestingly, sphingomyelin-phosphocholine with a myristic acid (C14:0) linking to a ceramide is detected. Both PC and sphingomyelin share a phophocholine functional group, yet it is not determined whether this sphingomyelin-phosphocoline can replace PC to be an important part of SOW structure. Additional sphingolipids are detected in relatively high amounts that include sphingosine, sphingosine-phosphate, sphingomyelin-phosphoethanolamine and dihexosylceramide. The latter is the predominant SOW glycosphingolipid that contains mainly saturated fatty acids (C14:0 and C16:0). Likewise, the prevalent glycolipid of *Paracoccidioides brasiliensis* is also a hexosylceramide (Hex-C18∶0-OH/d19∶2-Cer) that is linked to a longer C18:0 fatty acid (Longo et al., 2013). Further fatty acid composition analysis of SOW lipids reveals that palmitic acid (C16:0) and stearic acid (C18:0) are the two major components. These two fatty acids are commonly present in bilayer membrane. Notably, fatty acid composition of whole SOW lipids is different from the major glycosphingolipid. Taken together, these results suggest that the biosynthesis and metabolic enzymes in the SOW glycolipid synthesis pathways have a preference of shorter fatty acids (C14:0 and C16:0 compared to C18:0). Only few amounts of unsaturated fatty acids are detected in SOW lipids (**Table 2**). Biological membrane fluidity is highly regulated for cells to acclimate growth at different temperature conditions (Thompson and Nozawa, 1984). Temperature-induced alteration of fatty acid compositions for microbes has been reported (Hung et al, 1995). Cells grown at higher temperature tend to contain higher amounts of long chain and saturated fatty acids that have higher melting points compared to short chain and unsaturated fatty acids (Hung et al., 1995; Knothe and Dun, 2009). High content of saturated fatty acids may be beneficent for *Coccidioides* to grow at 39°C of the culture condition of the parasitic phase.

Fungal cell wall is an excellent reservoir of antigenic components that can contribute to the interactions between fungi and their hosts. The host attempts to recognize the microbial pathogen and inhibit its growth and dissemination, whereas the pathogen tries to subvert recognition and suppress host responses. SOWgp is predicted to be a GPI-anchored protein that is identified as a major antigenic glycoprotein located on SOW (Cole et al., 1988, Hung et al., 2000 and 2002). SOWgp elicits both humoral and cell-mediated immune responses in *Coccidioides* patients and mice that are vaccinated with formalin-killed spherules (Cole et al., 1988 and Hung et al., 2000). Mice vaccinated with bacterial-expressed recombinant SOWgp protein combined with the complete Freud’s adjuvant and other adjuvant systems gave non-consistent protective efficacy from 0 to 50% survival in a murine model of pulmonary coccidioidomycosis, depending on vaccine doses, adjuvants and mouse strains (Hung et al., 2000; Cox and Magee, 2004; data not shown). The protective capacity and immune regulation role of SOWgp is still elusive. Notably, the *sowgp* mutant strain created by targeted gene replacement shows partial loss of virulence (Hung et al., 2002). These findings suggest that beside the SOWgp, other components in the SOW may play an important role in modulation of the immune response to *Coccidioides* infection.

*Coccidioides* SOW lipids can modulate immune response is an appealing idea. PMNФs are the main cells recruited to the infection sites after C*occidioides* challenge. PMNФs are thought to play double-edged swords against many microbial infections. Early infiltration of PMNФs may facilitate microbial killing. On the other hand, mass infiltration of PMNФs may contribute to tissue damage (Kaplan and Radic, 2012). Endospores released from mature spherules trigger an influx of neutrophils to the *Coccidioides* infection sites. It has been showed *in vitro* that neutrophils can inhibit the growth of spherules initials and endospores (<10 μm) (Drutz and Huppert, 1983; Lee et al., 2015). Apparently, PMN*Ф*s are also essential in the early stage of vaccine-induced immunity, as depletion of PMN*Ф*s using a specific anti-Ly6G mAb renders the protective efficacy of the live, attenuated vaccine against pulmonary *Coccidioides* infection in mice (Hung et al, 2014). Interestingly, our data show that SOW lipids suppress PMNФs killing of *Coccidioides* arthroconidia. For the *in vivo* evaluation of the immune suppressive activity of SOW lipids, we used a murine model of subcutaneous *Coccidioides* spp infection. The subcutaneous infection route is not a common portal of entry for coccidioidomycosis, but it allows the delivery of a larger and more consistent dose of arthroconidia and results in significant fungal burden in the hypodermis but not dissemination of the pathogen to multiple vital organs (Hung et al., 2016). In this model, SOW lipid-treated mice develop disseminated disease to both the lungs and spleen after 20 days post-challenge while the mock treated mice mainly had fungal burden in the skin. These data confirm that SOW lipids suppress protective immunity and promote fungal dissemination. We early have reported that *Coccidioides* parasitic cells can release a soluble molecule(s) in culture medium to suppress nitric oxide production in bone marrow derived macrophages (Gonzalez et al., 2011). The work to explore whether SOW lipids can suppress NO production and other immune mechanisms is under way.

## Data Availability Statement

The raw data supporting the conclusions of this article will be made available by the authors, without undue reservation.

## Ethics Statement

The animal study was reviewed and approved by UTSA and handled according to guidelines approved by IACUC.

## Author Contributions

CP-J did all the lipid extraction procedures and the chemical characterization. MdP-JA designed and did the biological activity evaluation experiments. PA-M helped in the HPL/MS analysis and with CP-J did the compound identification. C-YH designed and analyzed the experiments. NC-L helped with the mice experiments. GC designed and analyzed the experiments. All the authors contributed to the article and approved the submitted version.

## Funding

This work was supported by the National Institutes of Health Grants R01-AI071118A (to GC and to MJ-A) and R01AI135005 (to C-YH) and Universidad de Antioquia to CP-J. and MJ-A).

## Conflict of Interest

The authors declare that the research was conducted in the absence of any commercial or financial relationships that could be construed as a potential conflict of interest.

## Supplementary Material

Supplementary Figure 1 Western blot showing the lack of the SOWgp protein.

Supplementary Table 2 CD11+Ly6G+ positive cells after the Ficoll-Paque density gradient.

Supplementary Figure 3
*Coccidioides* SOW lipids did not affect the viability of C57BL/6 and DBA/2J neutrophils.

Supplementary Table 3 Annexin V and CFDA staining of neutrophils treated with different concentrations of SOW lipids.
