# Supplementary material for: Lipid Secretion by Parasitic Cells of Coccidioides Contributes to Disseminated Disease
Source: Front Cell Infect Microbiol. 2021 May 13;11:592826. doi: 10.3389/fcimb.2021.592826 (PMC8155295; doi:10.3389/fcimb.2021.592826)

Supplemental material 1:

FIGURE S1.
Western blot showing the lack of the SOWgp protein

Line 1: lipids extracted from SOW

Line 2: SOW

MW 1 2

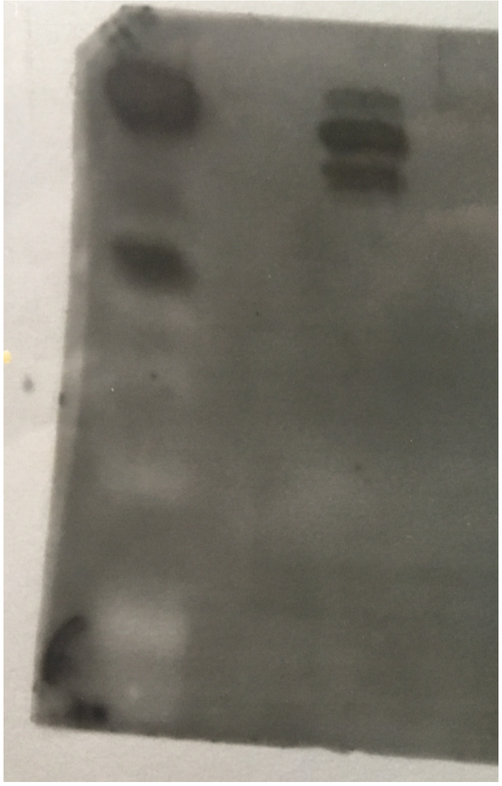

Supplement: Supplementary Figure 1 — Western blot showing the lack of the SOWgp protein. [file DataSheet_1.pdf]
