# Supplementary material for: Lipid Secretion by Parasitic Cells of Coccidioides Contributes to Disseminated Disease
Source: Front Cell Infect Microbiol. 2021 May 13;11:592826. doi: 10.3389/fcimb.2021.592826 (PMC8155295; doi:10.3389/fcimb.2021.592826)
Supplement: Supplementary Table 2 — CD11+Ly6G+ positive cells after the Ficoll-Paque density gradient. [file DataSheet_2.pdf]

Supplemental material 2:

Confirmation of the neutrophils isolated from the mice via flow cytometry:

After the Ficoll-Paque density gradient, we analyzed by flow cytometry the cells recovered. In the first experiment we stained with: CD11b+, Ly6G+ and Cd11+Ly6G+ antibodies. In the second experiment we used: CD45+, Ly6G+; Mac3+, CD11b+Ly6G+, CD11b+Mac3+, CD11b+Ly6g-, CD11b+Mac3-. In the third experiment the cells recovered were analyzed as follow: CD45+, CD11b+ IN CD45+, CD11b+Ly6G+, CD11b+ Mac3+, Ly6G+ IN CD45 and mac3 In CD45+. In the experiment number 4, the cells were stained withCD45+, CD11b+Ly6G+and CD11b+Mac3+. The medians of percentage of CD11+Ly6G+ positive cells is shown in TABLE S2.

TABLE S2. CD11+Ly6G+ positive cells after the Ficoll-Paque density gradient.

**CD11+Ly6G+ (Median of %)**	
**C57BL/6**	**DBA/2J**
60.6	49.7
