# Supplementary material for: Lipid Secretion by Parasitic Cells of Coccidioides Contributes to Disseminated Disease
Source: Front Cell Infect Microbiol. 2021 May 13;11:592826. doi: 10.3389/fcimb.2021.592826 (PMC8155295; doi:10.3389/fcimb.2021.592826)
Supplement: Supplementary Figure 3 — Coccidioides SOW lipids did not affect the viability of C57BL/6 and DBA/2J neutrophils. [file DataSheet_3.pdf]

Supplemental material 3:

The *Coccidioides* SOW lipids did not affect the viability of C57BL/6 and DBA/2J neutrophils as it was determined with CFDA and Annexin V staining by flow cytometry (FIGURE S3) The percentage of live cells was determined by CFDA positive and Annexin V negative; the percentage of early apoptotic cells was determined as Annexin V and CFDA positive cells and the percentage of late apoptotic cells was determined by Annexin V positive and CFDA negative cells. The results are shown as the median of percentage between the findings of the three experiments performed (TABLE S3).

FIGURE S3. *Coccidioides* SOW lipids did not affect the viability of C57BL/6 and DBA/2J neutrophils.

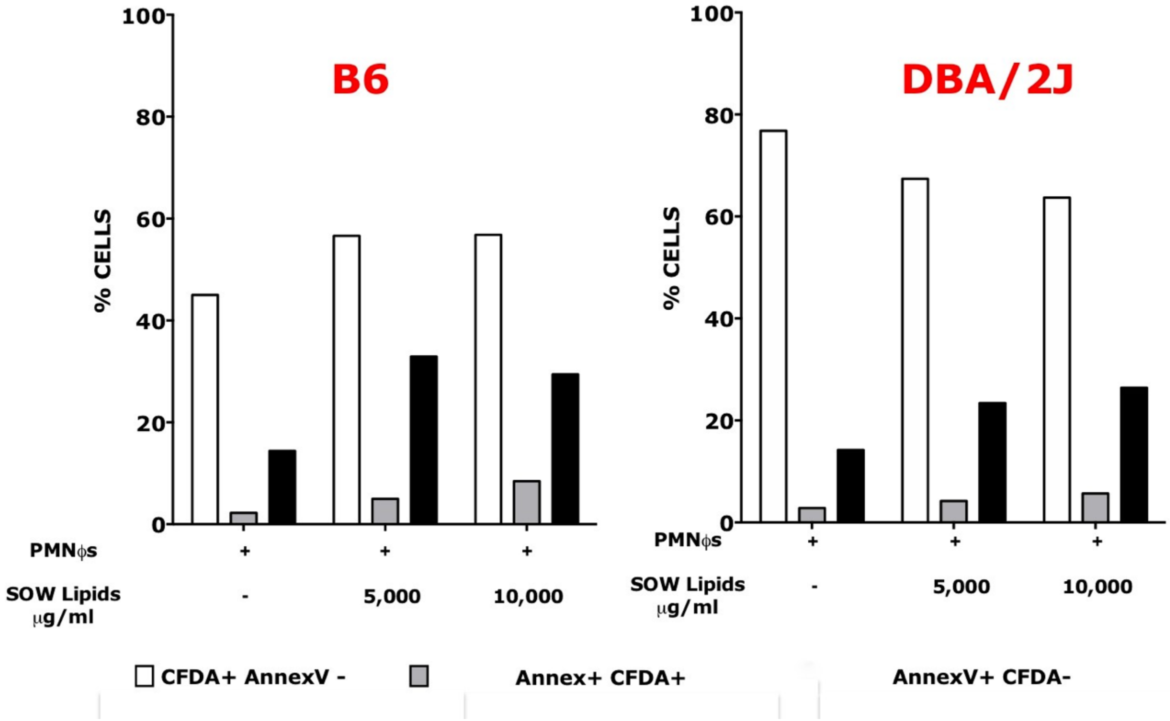

TABLE S3. Annexin V and CFDA staining of neutrophils treated with different concentrations of SOW lipids.

	ANNEXIN V+ (MEDIAN OF %)		ANNEXIN V + CFDA + (MEDIAN OF %)		CFDA + (MEDIAN OF %)	
	C57BL/6	DBA/2J	C57BL/6	DBA/2J	C57BL/6	DBA/2J
**Neutrophils**	11.31	16.5	4.44	3.45	77.05	74.95
**Neutrophils+1000ug/ml SOW lipids**	49.9	______	3.81	_____	66.7	
**Neutrophils+5000ug/ml SOW lipids**	42.1	20.8	4.98	4.59	56.6	70.8
**Neutrophils+10000ug/ml SOW lipids**	29.4	26.4	5.6	5.68	70.6	63.7
